# Metabolomic investigations in cerebrospinal fluid of Parkinson's disease

**DOI:** 10.1371/journal.pone.0208752

**Published:** 2018-12-10

**Authors:** Desiree Willkommen, Marianna Lucio, Franco Moritz, Sara Forcisi, Basem Kanawati, Kirill S. Smirnov, Michael Schroeter, Ali Sigaroudi, Philippe Schmitt-Kopplin, Bernhard Michalke

**Affiliations:** 1 Analytische BioGeoChemie, Helmholtz Zentrum München, Neuherberg, Germany; 2 Klinik und Poliklinik für Neurologie, Uniklinik Köln, Köln, Germany; 3 Klinik für Klinische Pharmakologie und Toxikologie, Universitätsspital Zürich, Zürich, Switzerland; 4 Institut I für Pharmakologie, Zentrum für Pharmakologie, Uniklinik Köln, Köln, Germany; 5 Analytische Lebensmittelchemie, Wissenschaftszentrum Weihenstephan, TU München, Freising, Germany; Weill Cornell Medical College in Qatar, QATAR

## Abstract

The underlying mechanisms of Parkinson´s disease are not completely revealed. Especially, early diagnostic biomarkers are lacking. To characterize early pathophysiological events, research is focusing on metabolomics. In this case-control study we investigated the metabolic profile of 31 Parkinson´s disease-patients in comparison to 95 neurologically healthy controls. The investigation of metabolites in CSF was performed by a 12 Tesla SolariX Fourier transform-ion cyclotron resonance-mass spectrometer (FT-ICR-MS). Multivariate statistical analysis sorted the most important biomarkers in relation to their ability to differentiate Parkinson versus control. The affected metabolites, their connection and their conversion pathways are described by means of network analysis. The metabolic profiling by FT-ICR-MS in CSF yielded in a good group separation, giving insights into the disease mechanisms. A total number of 243 metabolites showed an affected intensity in Parkinson´s disease, whereas 15 of these metabolites seem to be the main biological contributors. The network analysis showed a connection to the tricarboxylic cycle (TCA cycle) and therefore to mitochondrial dysfunction and increased oxidative stress within mitochondria. The metabolomic analysis of CSF in Parkinson´s disease showed an association to pathways which are involved in lipid/ fatty acid metabolism, energy metabolism, glutathione metabolism and mitochondrial dysfunction.

## Introduction

Parkinson´s disease (PD) is a severe neurodegenerative disease with a prevalence of 0.6% in 65 to 69 years old population that increases up to 3.5% in the population between 85 and 89 years old [1]. The clinical diagnosis relies on the typical cardinal symptoms: resting tremor, bradykinesia, and rigidity. Hallmark of pathophysiological events is the progressive loss of dopaminergic neurons, but symptoms appear when at least 60–80% of dopaminergic neurons are lost [2]. Up to date, early diagnostic biomarkers are lacking [3], therefore emerging interest is moving towards metabolic changes due to disease. Consequently, recent research is focusing on metabolomics to uncover early metabolic events in PD by the use of different analytical technologies.

LeWitt *et al*. applied ultrahigh performance liquid chromatography-mass spectrometry and gas chromatography-mass spectrometry for the analysis of plasma and cerebrospinal fluid (CSF) metabolome of 49 PD affected patients [4]. They identified prognostic plasma-biomarkers. Burté et al. identified, by MS-based techniques, 20 metabolites in human serum associated with PD and mild cognitive impairment [5]. The identified metabolites were mainly linked to the fatty acid oxidation pathway. A metabolic profiling approach was implemented for CSF by the use of NMR resulting in 15 metabolites, predominantly amino acids, which distinguished between PD and control [6]. The mouse brain metabolome of the disease manganism–a Mn-related Parkinsonian disease—was investigated by Fourier transform-ion cyclotron resonance-mass spectrometry (FT-ICR-MS) [7, 8], a MS-based technique that offers ultra-high resolution and mass accuracy. This technique allows for the concurrent detection of thousands of metabolites in a complex matrix [9] as well as the assignment of putative molecular formulas [10, 11] to each experimentally detected feature. Changes in amino acid, fatty acid, glutathione, glucose and purine/pyrimidine metabolisms were detected, showing an increase in oxidative stress and in inflammation markers [7, 8]. A recent review summarizes the results of metabolic profiling studies in Parkinson´s disease [12].

Generally, the metabolomics’ investigations can be targeted or non-targeted. A targeted investigation is restricted to a specific compound or a class of compounds whereas non-targeted analysis provides a comprehensive chemical characterization of a complex bio/chemical system [13]. The analysis by FT-ICR-MS is semi-quantitative and considers the intensity of each detected feature as a measure of their concentration. Therefore, this technique is suitable for the profile screening in a discovery oriented way. For the first time, to our knowledge, a non-targeted FT-ICR-MS was applied to get a not restricted deeper knowledge about the biochemical pathways involved in PD. A suitable liquid to reflect metabolomic changes in neurodegenerative diseases is CSF, which is in close contact to the brain [14, 15].

A machine learning approach sorted the most important compounds in relation to their ability to differentiate PD and control. An additional network analysis revealed mass differences of the selected, most important compounds for supplemental information about ongoing processes. Notably, the network analysis showed a connection to the tricarboxylic acid (TCA) cycle, to mitochondrial dysfunction, and increased oxidative stress within mitochondria. With this unique combination of metabolic profiling and network analysis, the investigations provided deeper insights into ongoing disease mechanisms and the major effectors of the disease. Especially oxidative stress (e.g. lipid peroxidation and changes in antioxidant compounds like glutathione) and neuroinflammation (e.g. arachidonic acid) are known to be involved in PD [16] and hence are the main focus of our investigation. Alterations may be due to primary disease processes as well as due to compensatory or reactive (e.g. inflammatory) mechanisms and epiphenomena.

## Materials and methods

### Chemicals

The purchased chemicals are: MeOH from CHROMASOLV LC-MS (Sigma-Aldrich, St. Louis, USA) and L-Arginine from Sigma Aldrich (>98% purity, St. Louis, USA).

### Study participants

A total of 126 CSF-samples were taken by standardized lumbar puncture at the Cologne University Hospital and were originally not intended for scientific use, but stored in the biobank of the hospital. The procedure of lumbar puncture was performed without problems and patients recovered quickly. Thirty-one of the CSF-samples were taken from patients diagnosed with PD and 95 samples derived from neurologically healthy controls (Table 1). The control patients underwent lumbar puncture after neurological symptoms (e.g. headache, dizziness) to exclude diseases of the central nervous system. Regarding the medication of the PD-patients, the patients are divided into 18 patients without any PD-specific medication, 11 patients with PD-specific medication (one or more of the following drugs: L-dopa, Madopar, Clarium, Sifrol/Pramipexol, Azilect, amantadine, and Artane), and two patients had electrodes for deep brain stimulation.

**Table 1:** Characteristics of PD and controls.

	Parkinson Patients	Healthy controls
Number of CSF-samples	n = 31	n = 95
Age (years)	65.5 ± 12.2	44.9 ± 17.3
Sex (f/m)	9/22	59/36
duration of disease (years)	0,87 ± 2,2	/

After lumbar puncture the samples remained at room temperature up to 6 hours for routine diagnostics. Subsequently, the samples were stored at -20±1°C temporary and later at -80±1°C until measurement. The count of erythrocytes was determined in a semi-quantitative manner in a counting chamber (negative = no erythrocytes, isolated < 5 erythrocytes/μL, + < 90 erythrocytes/μL, ++ > 90 erythrocytes/μL, +++ > 350 erythrocytes/μL, plentiful = overlying erythrocyte layers). Only samples with negative or isolated erythrocytes were involved in the study. The study was approved by the Ethics Committee of the University Cologne (09.12.2014, no. 14–364). All patients consented to scientific use of their CSF-samples.

### Sample preparation

Prior to FT-ICR-MS analyses a protein precipitation extraction (PPE) was performed. The protocol was adapted from Forcisi et al. [9]. The frozen CSF-samples were thawed on ice and vortex-mixed for 30 seconds prior to treatment. Ice-cold MeOH (320μl) was added to an 80 μL aliquot of each CSF sample. The samples were vortex-mixed for 30 s at room temperature and centrifuged at 18,900 g for 10 min at 4°C. The recovered supernatant was diluted (dil. factor: 1/70) in MeOH prior to FT-ICR-MS analysis.

### FT-ICR-MS measurement

Ultrahigh resolution mass spectra were acquired by means of FT-ICR-MS (Solarix, Bruker, Bremen, Germany), equipped with a 12 Tesla superconducting magnet (Magnex Scientific, Varian Inc., Oxford, UK) and an electrospray ionization (ESI) source (Apollo II, Bruker Daltonics, Bremen, Germany). An external calibration was performed by analysis of a 3 mg/L arginine solution in MeOH with calibration errors below 0.1 ppm. All measurements were performed in negative ionization mode and ion accumulation time of 300 ms for higher sensitivity. The injection flow rate was 2 uL/min for electrospray. Operating temperature was 180°C for rapid solvent evaporation inside the electrospray. The ESI nebulizer gas flow rate was 2 L/min and the dry gas flow rate 4 L/min. The spectra were recorded in a mass-to-charge-ratio (m/z) range of 123–1000. For the generation of each mass spectrum 300 scans were acquired. A time-domain transient of 4 MW size was produced for each acquisition, which yielded ultra-high resolution for all signals, which are of metabolomic interest.

### Data pre-treatment and statistics

The spectra were calibrated with an in-house calibration tool developed in Matlab (Release 2016a, The MathWorks, Inc., Natick, Massachusetts, US). The main principle is based on estimating the most probable calibration curve given the density map describing the behavior of a mass accuracy along the considered mass range. The extracted peaks were aligned within a 1 ppm tolerance window and stored in a data matrix [17]. The masses with a frequency below 10% were not considered during further data mining, the intensities of absent masses were set to zero in the related samples. We applied the in-house developed software *Netcalc* to remove potential spectral noise and isotope peaks. This software assigns molecular formulas to the aligned *m/z-peaks* based on a mass difference between the detected features [18]. Moreover, additional annotation was performed using the web server MassTRIX [19, 20] with *Homo sapiens* as reference organism. All annotations were stored in the original data matrix. The assignment of a molecular formula to each m/z values was performed by molecular formula propagation through mass difference networks (MDiN). Here, m/z features (nodes) were connected by mass differences (edges) with corresponding molecular formula labels (e.g. Δm/z = 14.01565 → ΔCH_2_) and random walks starting from known peaks updated the molecular formulas of yet unassigned m/z peaks. The network was optimized to correct conflicting relationships and to closely follow the intrinsic m/z-error distribution of a spectrum for which reason this so-called Netcalc-algorithm is considered as an unsupervised filter that reduces the data size and reveals an underlying biochemical network structure inside the data set [21]. In order to improve the efficiency of the classification (Parkinson versus controls) and reduce possible overfitting and noise, we preprocessed the entire dataset applying the ReliefF algorithm [22]. The algorithm identified a subset of variables (in total 243 masses) that was able to maximize the classification accuracy of the subsequent classification models. The features’ selection was based on the highest rank value attributed to each variable by the software. For each masses (stored in the S2 Table) we reported, the sensitivity, the specificity, the positive predictive value (PPV) and the negative predictive value (NPV). A sparse Partial Least Squares-Discriminant Analysis (sPLS-DA) was built in order to assign the respective metabolites for each class from the list of 243 masses. sPLS-DA imposes sparseness within the latent components to improve variables selection while performing simultaneous dimension reduction. A 7-fold cross-validation together with the receiver operating characteristic curve (ROC) was chosen to evaluate the classification performance. For this model, the Balanced Error Rate (BER) has been calculated to evaluate the performances. BER is appropriate in case of an unbalanced number of samples per class as it calculates the average proportion of wrongly classified samples in each class [23]. For the classification model we used the MixOmics package and for the values presented in S2 Table (sensitivity, specificity, PPV and NPV) we have used reportROC package (RStudio Version 1.0.136 – 2009–2016 RStudio, Inc.) Moreover, an Orthogonal Projections to Latent Structures-Discriminant Analysis (OPLS-DA) model was applied to have another classification model and to describe the orthogonal variance. The performance of the fit and the prediction were evaluated with the R^2^ and Q^2^ values. Moreover, we provide the p-value for Analysis of Variance of Cross-Validation Estimators. Those elaborations were done in SIMCA 13.0.3.0 (Umetrics, Umeå, Sweden).

For the most important metabolites we did an analysis of covariance (ANCOVA) testing the significance for the interactions of the factor (Parkinson vs. control) with age and also with gender. Then we calculated all the p-values (adjusted by Dunnett test) of the differences between Parkinson vs. control (being an unbalanced experimental design we chose to compare the least squares means) controlled by gender (listed in Table 2). The elaboration was done using the general linear model (GLM) analysis in SAS 9.4 (SAS Institute Inc., Cary, NC, USA).

**Table 2:** Most important neutral masses to distinguish between PD and controls with respective molecular formula, possible compounds assignment and mean intensity ± standard deviation (SD). The p-values are the result of the general linear model (GLM) adjusted with DUNNET.

Neutral mass	Theoretical molecular ion mass	molecular formula	Ion formula	Compound most probable in CSF	Mean Intensity ± SD control	Mean Intensity ± SD Parkinson´s disease	alteration in Parkinson´s disease	p-value
129.04261	128.03534	C_5_H_7_NO_3_	[C5H6NO3]^-^	5-Oxoproline	1.05E+06 ± 1.09E+06	1.5E+06 ± 1.27E+06	↑	0.0795
188.01433	187.00705	C_7_H_8_O_4_S	[C7H7O4S]^-^	p-cresol sulfate	5.74E+04 ± 3.23E+05	5.33E+05 ± 1.03E+06	↑	0.0002
186.06411	185.05684	C_7_H_10_N_2_O_4_	[C7H9N2O4]^-^	S-AMPA	1.49E+05 ± 4.69E+05	0.0E+0 0± 0.0E+0.0	↓	0.2695
192.06343	191.05616	C_7_H_12_O_6_	[C7H11O6]^-^	Quinic acid	6.30E+05 ± 1.02E+06	1.07E+06 ± 1.3E+06	↑	0.019
260.02032	259.01305	C_6_H_12_O_9_S	[C6H11O9S]^-^	D-Glucose-6-sulfate	4.1E+05 ± 8.18E+05	5.54E+05 ± 8.94E+05	↑	0.3258
163.09980	162.09253	C_10_H_13_NO	[C10H12NO]^-^	N-Acetylphenyl-ethylamine	1.5E+04 ± 1.48E+05	1.49E+05 ± 4.66E+05	↑	0.0578
210.07402	209.06675	C_7_H_14_O_7_	[C7H13O7]^-^	Sedoheptulose	1.31E+06 ± 1.14E+06	8.06E+05 ± 9.76E+05	↓	0.0701
268.07956	267.07228	C_9_H_16_O_9_	[C9H15O9]^-^	α-mannosylglycerate	1.32E+06 ± 1.49E+06	1.78E+06 ± 1.78E+06	↑	0.1406
172.14637	171.13910	C_10_H_20_O_2_	[C10H19O2]^-^	Decanoic acid	4.69E+04 ± 2.64E+05	2.38E+05 ± 6.42E+05	↑	0.0116
188.14116	187.13389	C_10_H_20_O_3_	[C10H19O3]^-^	10-Hydroxydecanoic acid	1.67E+04 ± 1.64E+05	1.76E+05 ± 5.5E+05	↑	0.0129
234.16207	233.15480	C_15_H_22_O_2_	[C15H21O2]^-^	Valerenic acid	8.67E+05 ± 1.06E+06	1.23E+06 ±1.19E+06	↑	0.1567
304.24043	303.23316	C_20_H_32_O_2_	[C22H31O2]^-^	Arachidonic acid	4.01E+05 ± 8.42E+05	9.15E+05 ± 1.52E+06	↑	0.0494
306.25612	305.24885	C_20_H_34_O_2_	[C20H33O2]^-^	Dihomo-γ-linolenic acid	6.61E+05 ± 1.28E+06	8.62E+05 ± 1.19E+06	↑	0.4911
622.55332	621.54604	C_39_H_74_O_5_	[C39H73O5]^-^	DG (36:1)	1.73E+06 ± 2.23E+06	8.17E+05 ± 1.62E+06	↓	0.0677
747.61377	746.60650	C_42_H_86_NO_7_P	[C42H85NO7P]^-^	PC/PE	1.58E+06 ± 1.29E+06	1.09E+06 ± 1.24E+06	↓	0.0291

Additionally, a mass difference enrichment analysis (MDEA) was performed following Moritz *et al*. [24]. The list of mass difference building blocks (Δm), investigated for enrichment with PD-markers, was obtained from the supporting material of the same publication [24]. The network was reconstructed on the full set of features with molecular formula assignment, including the 243 differentially regulated features (features selected with the ReliefF algorithm). The complete detected metabolome is assumed to provide the substrates for the (bio-) synthesis of these 243 disease markers. MDEA tests which Δm’s (biochemical reactions) connect markers to the remaining metabolome and therewith highlights probable reactions of biomarker synthesis [24, 25]. Fisher’s exact test was used to evaluate enrichment, resulting in Z-Scores and p-values. The Z-scores of Z ≈ 2 and Z ≈ 2.5 relate to p ≈ 0.05 and p ≈ 0.01, respectively. This way, MDEA addresses the differential usage patterns of molecular building blocks in the biosynthesis of PD- and Control-markers.

## Results

**Fig 1:**
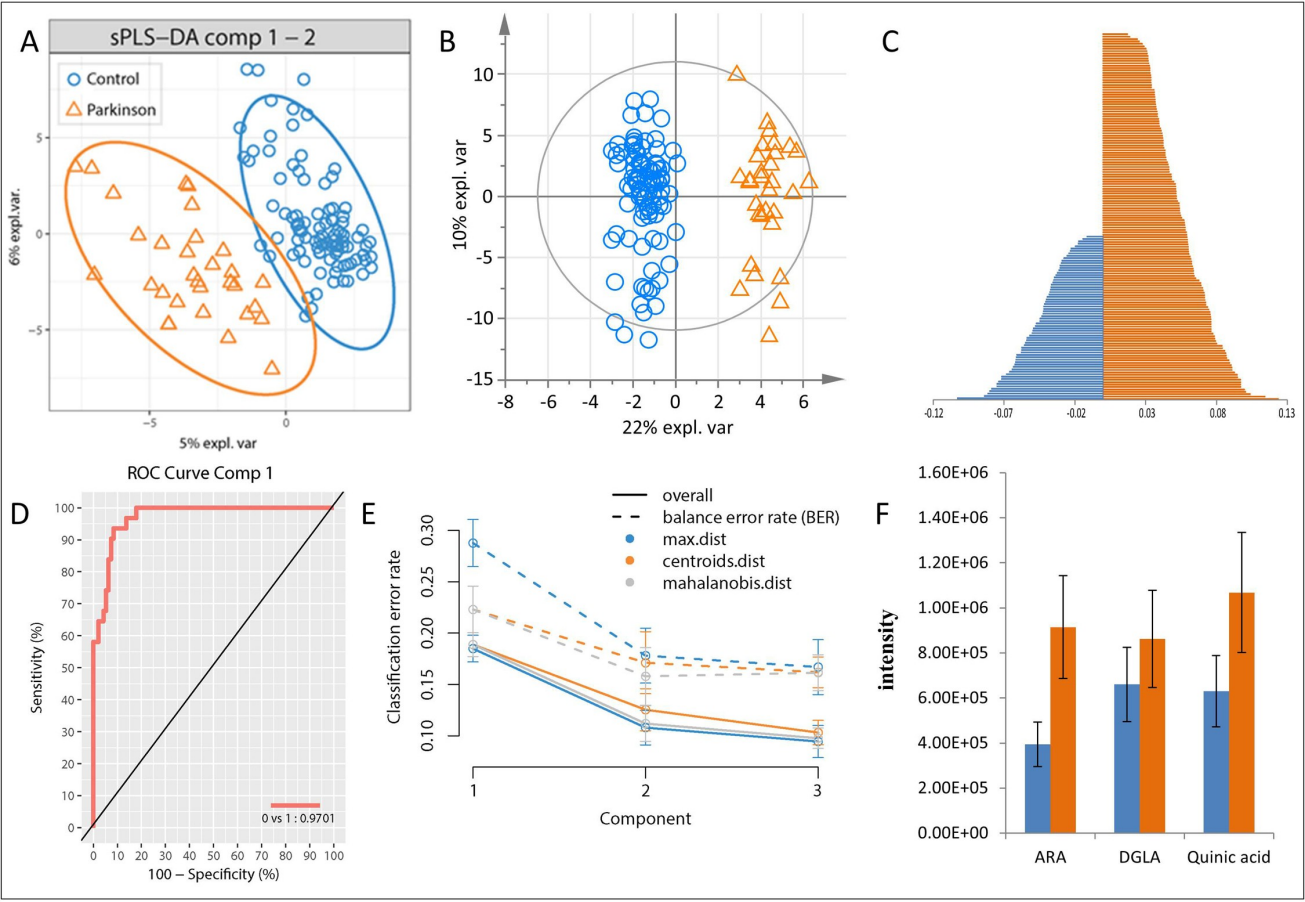
The implemented statistical analysis models. A) sPLS-DA and B) OPLS-DA both validated with 7 fold cross-validation, C) compounds, which significantly distinguished PD from controls, D) and E) represented the area under the Receiver Operating Characteristic (ROC) curve and the classification error rates by which the number of components was tuned (7 cross-validation), F) compared intensities of selected lipids. expl. var., explorative variance.

The metabolic profiles of 31 Parkinson-patients and 95 controls were analyzed by FT-ICR-MS. A features selection was performed after the annotation of elemental formulas of the respective m/z-values in the generated data matrix. The feature selection excluded a part of the data noise and the information not related to the study design (Parkinson vs. control). The subset of variables calculated with such algorithm was reduced to 243 m/z values. Consecutively, we wanted to test if the list of 243 m/z was capable to separate the two groups under investigation. We built a sPLS-DA analysis (Fig 1A) and an OPLS-DA (Fig 1B). Both were able to separate controls from diseased individuals [26]. This separation was even clearer with the OPLS-DA analysis, showing good values for the fitting and prediction (R^2^Y(cum) = 0.98 and Q^2^(cum) = 0.53. The cross validation Anova gave a p-value < 0.0001). The OPLS-DA explained 32% of variance with the first two components (the sPLS-DA explained with the three main components 16% of variance). The values of the Balanced Error Rate (BER) for the sPLS-DA model are in the supplementary S5 Table. Moreover, we have observed that in both plots (1A and 1B) the youngest patients do not cluster all together. In the clustering, we didn’t find any trends for the age, meaning that the factor that drives the separation is related with the health status of the person. Among the 243 masses, we found 81 metabolites with decreasing and 162 metabolites with increasing signal intensities for PD samples relative to controls (Fig 1C), according with both models (S2 Table). The highest loadings values were chosen as the most explicative variables in the class separation. Fig 1D represents the ROC curve calculated from the first component of the sPLS-DA analysis. The ROC curve, calculated on the 243 subset of masses confirmed that this list could be optimal for the discrimination of the two groups. Moreover, Fig 1E represents the performance plot. It is based on t-tests for significant difference in the mean error rate between components. The error rate after the second components seems to be stabilized. Therefore, two components are sufficient to achieve a good performance.

From the subset of metabolites we investigated only the possible assigned with the KEGG database (in total are 32). Among those 32 candidates, we presented in Table 2 the most relevant from a biological point of view (all possible assignments for these most relevant metabolites are listed in S3 Table). Most of the affected compounds derived from the compound class of lipids (decanoic acid, 10-hydroxydecanoic acid, arachidonic acid, dihomo-γ-linolenic acid, diacylglycerol (DG), phosphatidylcholine (PC) and phosphatidylethanolamine (PE)). Additionally, sugar derivatives and carboxylic acids were affected.

Moreover, an ANCOVA analysis was performed to evaluate the influence of age and gender on the results. The analysis did not reveal any significant interactions between age and the main factor (Parkinson vs. control) for each of the metabolites presented in Table 2. The only significant interaction was found for gender for the Arachidonic acid (p = 0.003).

**Fig 2:**
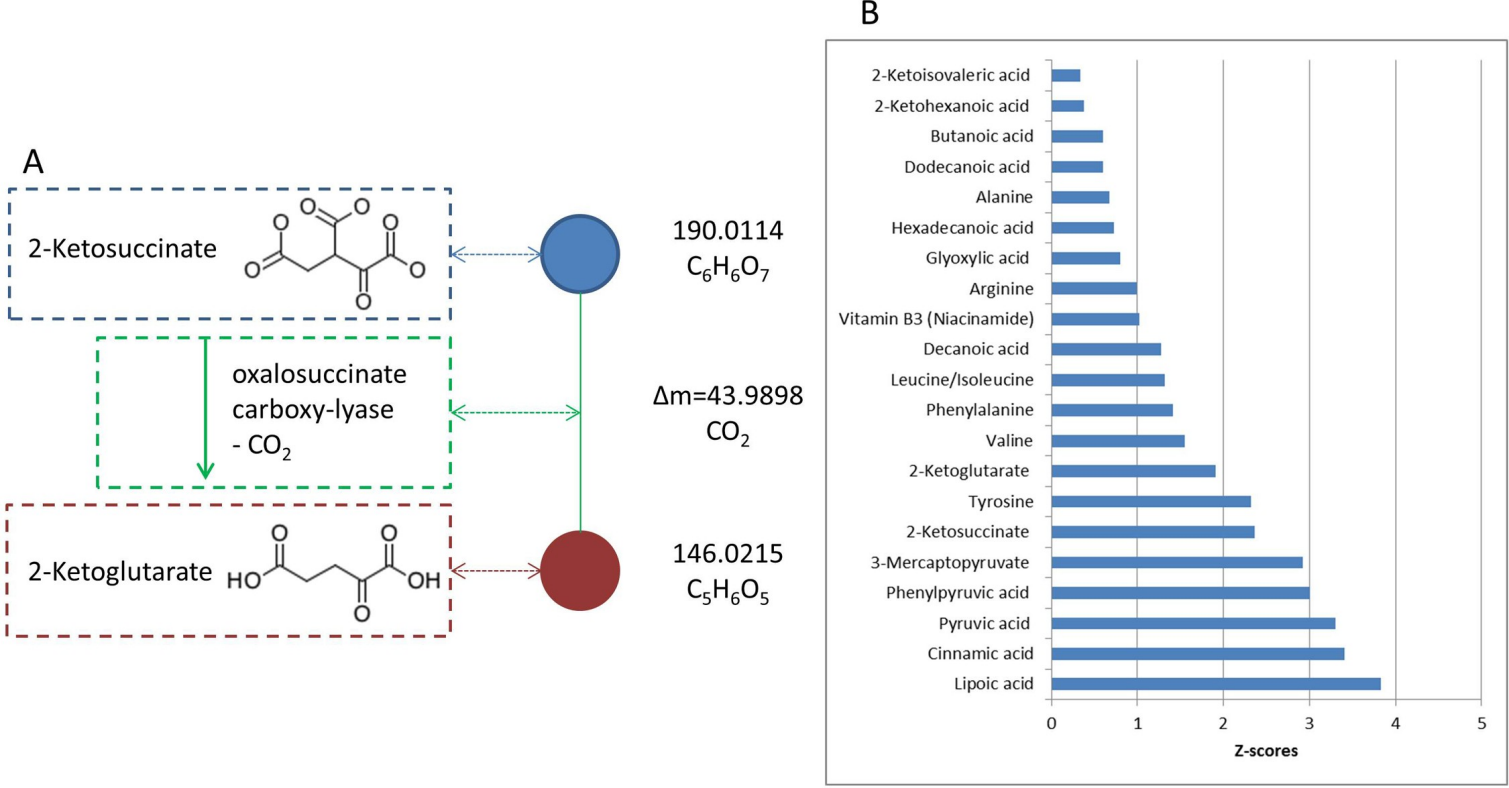
A) theory behind the network analysis shown for a specific example, B), over-represented Δm for PD as characterized by respective Z-scores.

Based on the feature selection and the classification models results, the entire dataset (S1 Table) was analyzed by a network approach. The network was build up by connecting exact m/z-features (nodes) using mass differences (Δm) that were derived from biochemical reactions as demonstrated in Fig 2A for the conversion of 2-Ketosuccinate to 2-Ketoglutarate. The Δm’s are characterized by Z-scores, which represent the increase or decrease of a Δm’s occurrence with significantly regulated metabolic features (all the z-scores are listed in S4 Table). Fig 2B illustrates the over-represented Δm s in PD. Different compounds, involved in the cellular respiration processes namely the TCA cycle, were observed increased in PD. These compounds, which are part of the TCA cycle like α-ketoglutarate and pyruvate, were increased, but also substrates for the synthesis of compounds of the TCA cycle like amino acids and break-down products of several TCA-compounds were found over-represented in PD. Additionally, the compounds lipoic acid and vitamin B3 are over-represented MDBs, which are important for the function of pyruvate dehydrogenase.

## Discussion

In this study, we investigated the metabolic profile of CSF-samples from PD patients and controls. Our approach used CSF-samples to investigate the changes due to disease. CSF is a suitable biofluids to investigate changes in neurodegenerative diseases, because it is in close contact to the brain. This biofluids is in direct contact to the extracellular space of brain parenchyma [14] and therefore metabolic changes within brain are likely to be reflected in CSF [15]. In contrast to more common targeted analysis like LC-MS, this non-targeted FT-ICR-MS analysis can be used to create new hypotheses, to evaluate as much compounds as possible, and to compare results with other metabolomic investigations already done in PD. The main aim of non-targeted determination by FT-ICR-MS is the spectral profile comparison of healthy and diseased state. Targeted approaches need a prior hypothesis, e.g. a specific compound class to determine, since only a part of the metabolome can be quantified. This application has major advantages when selected compounds have to be measured and can be used as a follow up to non-targeted methods. It has to be clear, that none of the existing techniques can cover all metabolites [27]. Therefore, to get a complete and comprehensive metabolite overview it is necessary to use several techniques and methods to cover as much metabolites as possible.

As hypothesized, we revealed several altered metabolites in PD as compared to controls. Specifically, metabolites belonging to the lipid/ fatty acid, glutathione, and energy metabolism showed a strong shift. Especially the increased level of the fatty acid arachidonic acid is associated with increased oxidative stress and neuroinflammation [28–30].

Age and gender are important factors in metabolic balance [31–33]. The patient groups investigated within this study are not matched for age and gender. Therefore, an ANCOVA was performed to evaluate the influence of both factors on the differentiation of PD and control-patients. For the parameter age we couldn´t find any influence on the most significant metabolites detected in the CSF samples. In contrast to age, the differentiation between PD and control for arachidonic acid is also related to the gender. The interaction between the gender and the variable (PD vs. control) is significant (p = 0.003). A gender influence on arachidonic acid concentration was also ascertained by a meta-analysis of 51 publications. This gender influence was not true for other investigated fatty acids [34]. Moreover, 11 PD-patients received PD-specific medication, 2 patients had electrodes and 18 patients didn´t have a medication at the time point of sample taking. Since medication of patients is too diverse this covariate was not taken into consideration.

Statistical models were applied to the achieved mass spectra for calibration. All the models gave an overall agreement, isolating a common list of important masses altered in the two different groups. The statistical analysis models sPLS-DA and OPLS-DA confirmed the good group separation according to principal components 1 and 2. Moreover, the area under the curve (ROC) showed also a high performance of the classification model, since it was calculated based on the predicted scores [23]. Additionally, the statistical analysis provided an insight into the biological characterization of PD, and the consequent confirmation of some biomarkers in the literature which are discussed in the following paragraphs.

### Glutathione metabolism

5-Oxoproline is an oxidation product and therefore elevated levels are a sign of increased oxidation. It is also associated with oxidative stress [35]. 5-Oxoproline is a part of the γ-glutamyl-cycle and thus it is implicated in glutathione (GSH) -metabolism. GSH is a protective compound against oxidative stress by oxidizing to glutathione disulfide (GSSG) with simultaneous reduction of H_2_O_2_ [36]. A higher rate of GSH-biosynthesis is associated with decreased oxidative stress but also with higher need to remove reactive oxygen species (ROS). GSH is depleted with increasing age [37] and in neurodegeneration, especially decreased values were found in substantia nigra [38–40] and CSF [41] of PD-patients. In contrast to this, increased levels of GSH have been found in an early stage of disease, possibly for protection against further oxidative stress [37, 42]. Increased concentrations of 5-oxoproline were found in plasma of PD-patients [43]. Additionally, Wu et al. [44] found an increased urinary excretion of 5-oxoproline to be associated with a reduced availability of cysteine and glycine and hence reduced GSH-biosynthesis in vivo. A reduced GSH-biosynthesis is followed by accumulation of ROS after a short time and causes neurodegeneration. Although we did not measure GSH itself, the increased 5-oxoproline content we have found in PD seems to be associated to protection against oxidative stress in an early disease progress (duration of disease: 0.87 years).

### Energy metabolism

Metabolites belonging to the energy metabolism were found within our study. We discovered increased intensities of D-glucose-6-sulfate and α-mannosylglycerate and decreased intensity of sedoheptulose in CSF of PD-patients. The metabolite α-mannosylglycerate is part of the fructose and mannose metabolism, two compounds which were also found to be increased in CSF of PD-patients [45]. These metabolites are also linked to glycolysis [46], which is increased in conditions of oxidative stress to suppress oxidative phosphorylation in mitochondria [47]. In concordance with these increased concentrations of metabolites of the fructose and mannose pathways, Ahmed et al. found another metabolite linked with this pathway. Increased concentrations of sorbitol were found in plasma of PD-patients. Additionally, Michell et al. identified increased intensity of several monosaccharides in serum [48]. The used method was not able to further differentiate in specific monosaccharides. Moreover, a study investigating especially the changes of energy metabolism in dopaminergic cells after exposure to environmental/ mitochondrial toxins (model for PD) was performed [49]. They found increased concentrations of sedoheptulose and the hexoses glucose and myoinositol. Although the direction of some shifted metabolites is inconclusive comparing the studies, a clear hint to changes in the energy metabolism due to PD is given and needs further investigation.

### Fatty acids and lipid metabolism

Our investigations showed several fatty acids to be altered in PD. Quinic acid, decanoic acid, 10-hydroxydecanoic acid, valerenic acid, arachidonic acid, and dihomo-γ-linolenic acid were found with increased intensities in PD. The lipid metabolism compounds DG, PC, and PE had decreased intensities in PD. Medium and long chain fatty acids (5-dodecanoate, 3-hydroxydecanoate, docosadienoate, and docosatrienoate) were also found increased in biofluids (plasma, CSF) of PD-patients by use of non-targeted metabolomic approaches [4]. Other studies also found alterations in fatty acid composition, although with decreasing intensity in PD. Trupp et *al*. found decreasing C16 and C18 fatty acids in plasma of PD patients [43] and Michell et *al*. decreased amount of octenoic acid in serum [48] indicating a possible inverse behavior of fatty acids in CSF and serum/plasma.

Especially DGLA and arachidonic acid (ARA) were investigated several times in various body fluids. Both compounds are polyunsaturated fatty acids (PUFA) present in human brain. PUFAs are vulnerable to oxidative stress due to lipid peroxidation [28, 50]. DGLA can form either anti-inflammatory compounds or pro-inflammatory ARA. Additionally, ARA is bound to membranes in brain; it is released enzymatically upon inflammation [51]. We found an increased content of DGLA and ARA in CSF of PD, which implies an elevation in pro-inflammatory characteristics. The enzymatic oxidation of ARA forms multiple pro-inflammatory metabolites [51]. A study investigated the development of the prostaglandins (PG) PGE_1_ and PGE_2_ (anti-inflammatory/ pro-inflammatory) after dietary DGLA intake in rat plasma [52]. The PGE_1_-level and the PGE_2_-level increased after DGLA-administration, but PGE_1_ increase was much higher. This finding is linked to increased anti-inflammatory capacity, at least at the beginning of disease. The ARA metabolism is strictly regulated in normal brain, but any misbalance due to neuroinflammation or oxidative stress can increase ARA metabolism in the brain and can finally cause neurodegeneration [53]. Julien *et al*. [54] investigated the fatty acid profile in postmortem brain of PD and in parkinsonian monkeys by gas chromatography. They focused on fatty acids in the cortex of the brain and found a significant elevation of ARA in humans and in monkeys after administration of the drug levodopa. ARA signaling was also found to be increased in a rat model with PD [30]. Thereby, an up-regulation of the cytosolic phospholipase A_2_ was found in cortex and putamen in affected rats, which is associated with elevated neuroinflammation in the brains of diseased subjects. A disease-orientated investigation studied the metabolic changes in rat brain after an intravenous Mn-injection. K. Neth *et al*. [7] found decreased DGLA-levels and nine other fatty acids and an increase in the lipid mediators PGB_1_, 15-(S)-HETE and Resolvin D2, which are associated with inflammation. Our investigation showed also a significant increase of DGLA and ARA in PD (Fig 1F), which can be associated with neuroinflammation and oxidative stress. Although our results showed no marked increase in the pro-inflammatory lipid mediators, arachidonic acid seems to have a higher release from membranes due to inflammatory processes.

Additional information about reactions was provided by MDEA. The MDEA analysis showed reactions with malondialdehyde (MDA)-production over-represented in PD. A direct detection of MDA by FT-ICR-MS is impossible because of too small molecular weight and the labile character of the compound. MDA is a marker for oxidative stress and a break-down product of PUFAs. Therefore, MDA is also involved in lipid peroxidation. Several studies identified MDA as marker of PD [55]. They found increased MDA levels in plasma in an early and late disease stages. Significantly increased MDA levels in PD compared to controls were also found in erythrocytes [56] and in plasma [57]. A recent review gives an overview of the MDA-metabolism [28].

### Mitochondrial dysfunction

**Fig 3:**
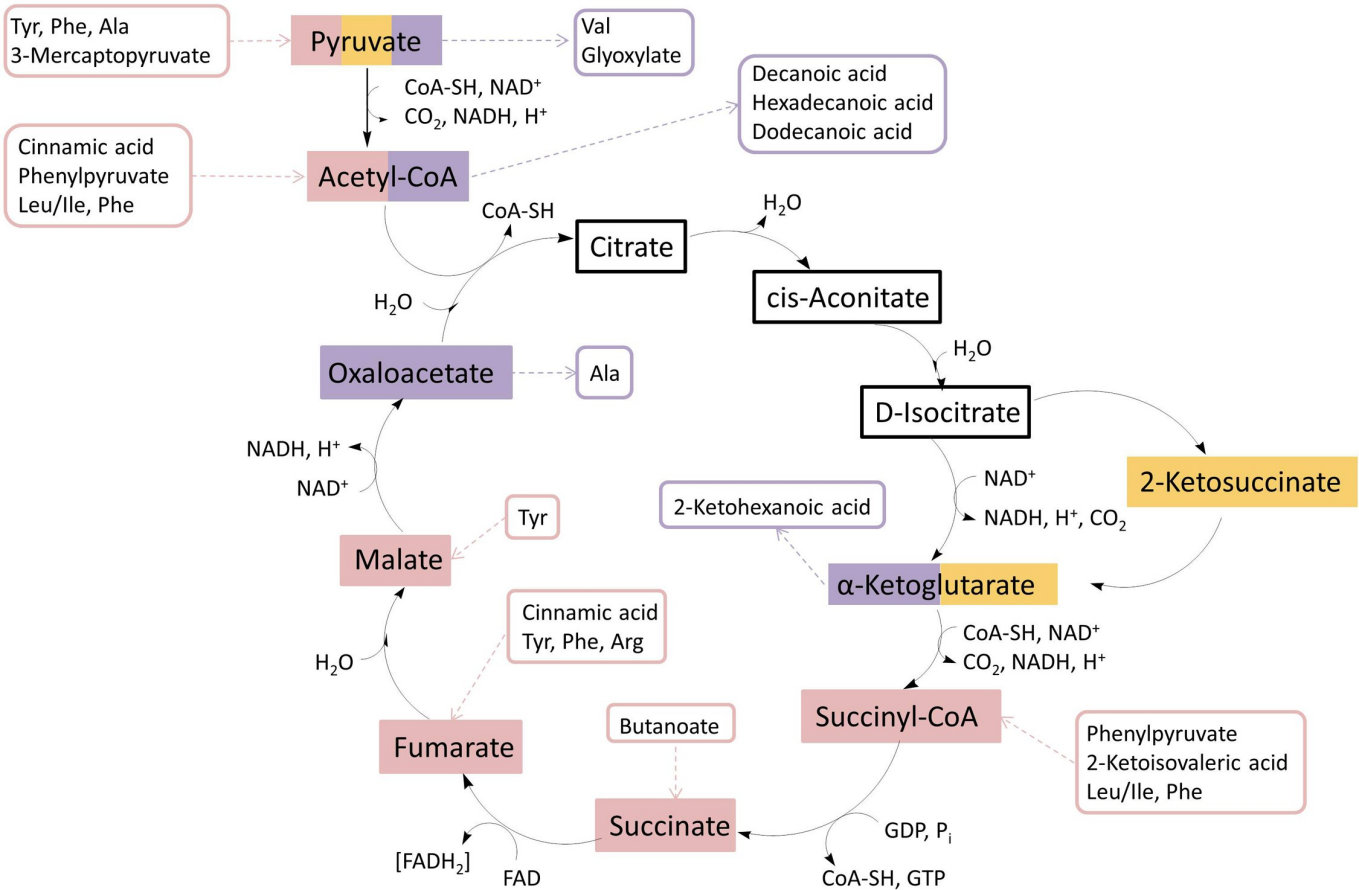
Over-represented metabolites within the TCA cycle. Metabolites directly found with network analysis are colored orange. Metabolites indirectly found either by substrates for synthesis of the respective compound (colored red) or by break-down products of the respective compound (colored purple).

Mitochondrial dysfunction is known to be involved in PD [58, 59]. Mitochondria produce the majority of cellular energy by oxidative phosphorylation. The 243 masses which differentiated controls and PD patients were further analyzed by MDEA to get an insight into processes involved in the disease. MDEA connected the masses within a network with specific mass differences explaining biochemical reactions and compared the abundance of each biochemical reaction in controls and PD. Our results showed over-represented metabolites associated with the TCA-cycle, as illustrated in Fig 3. We found compounds of the TCA-cycle over-represented (colored orange in Fig 3), compounds which are mandatory to synthesize compounds of the TCA-cycle (colored red in Fig 3) and also break-down products of several TCA-cycle compounds (colored purple in Fig 3). Mitochondrial dysfunction is primarily caused by ROS generated within mitochondria, but also metabolic dysregulation [60, 61]. Various metabolomic studies found altered metabolites of the TCA cycle within analysis. An increased concentration of malate in plasma of PD-patients was found by [43] and an increased concentration of citrate in CSF by [6]. In contrast to these observations, Ahmed et al. found decreased levels of several TCA metabolites (citrate, malate, succinate, and isocitrate) in plasma [62]. The inconsistent results may be due to differing sample matrix, sampling, storage and methods used and need further clarification. Apart from the compounds within the TCA cycle, different proteins, metals and other metabolites are needed for the synthesis and function of mitochondrial enzymes. Key nutrients are iron, manganese, copper, zinc, vitamin B3 and lipoic acid [63]. The metals are important central atoms in proteins, lipoic acid and vitamin B3 are important for the function of pyruvate dehydrogenase; the enzyme catalyzes the oxidative decarboxylation of pyruvate. An analysis of CSF with capillary zone electrophoresis hyphenated with inductively coupled plasma mass spectrometry shows the compounds fumarate, malate, oxaloacetate, α-ketoglutarate, citrate and NAD, which a part of the TCA-cycle, to be associated with manganese [64]. A change of associated transition metal to the metabolites could be an amplifying factor for increased oxidative stress in PD and also for mitochondrial dysfunction. A dysregulation of the metals iron, copper, manganese, and zinc was already found within these sample-set [65]. Thereby a dysregulation of specific ratios between different mass fractions of these metals were found to be significantly different in PD and controls. A correlation of shifted metal concentrations and metal ratios with TCA cycle compound could be beneficial to get further information regarding the relationship between metals and metabolites.

## Conclusion

In conclusion, the pipeline we used gave us an understanding of the unknown space investigated. We found several metabolites in CSF of PD and controls by using the untargeted metabolomics technique FT-ICR-MS. Due to the use of multivariate statistical analysis a differentiation between PD and controls was possible. Especially metabolites of the lipid metabolism showed up to be affected due to disease. Moreover, indices of mitochondrial dysfunction and alterations of the energy metabolism were found in PD. More research effort should be directed to targeted approaches to unravel the lipid metabolism pathways affected in PD. Additionally, correlation of metal-analysis with TCA cycle products may enable further insights into disease mechanisms.

## Supporting information

S1 Table Raw data of non-targeted metabolomic investigation in CSF. (XLS)

S2 Table Important masses detected with the ReliefF features selection algorithm. (XLS)

S3 Table Most important neutral masses to distinguish between Parkinson´s disease and controls with all possible assignments to respective masses. (DOC)

S4 Table Summary of network analysis. (XLS)

S5 Table Balanced Error Rate (BER). (XLS)

## Abbreviations

ARA: arachidonic acid
DG: diacylglycerol
DGLA: dihomo-γ-linolenic acid
ESI: electrospray ionization
ETC: electron transport chain
FT-ICR-MS: Fourier transform-ion cyclotron resonance-mass spectrometry
GSH: glutathione
GSSG: glutathione disulfide
MDA: malondialdehyde
MDB: mass difference building block
MDEA: mass difference enrichment analysis
MDiN: mass difference network
m/z: mass-to-charge-ratio
OPLS-DA: Orthogonal Projections to Latent Structure-Discriminant Analysis
PC: phosphatidylcholine
PD: Parkinson´s disease
PE: phosphatidylethanolamine
PG: prostaglandin
PPE: protein precipitation extraction
PUFA: polyunsaturated fatty acid
ROC: receiver operating characteristic
ROS: reactive oxygen species
sPLS-DA: sparse Partial Least Square-Discriminant Analysis
SOD: superoxide dismutase
TCA: tricarboxylic acid
