# Supplementary material for: Metabolomic investigations in cerebrospinal fluid of Parkinson's disease
Source: PLoS One. 2018 Dec 10;13(12):e0208752. doi: 10.1371/journal.pone.0208752 (PMC6287824; doi:10.1371/journal.pone.0208752)
Supplement: S3 Table — (DOC) [file pone.0208752.s003.doc]

Metabolomics investigations in cerebrospinal fluid of Parkinson´s disease

Supporting Information

**Authors:** Desiree Willkommen^1*¶^, Marianna Lucio^1*¶^, Franco Moritz^1^, Sara Forcisi^1^, Basem Kanawati^1^, Kirill S. Smirnov^1^, Michael Schroeter^2^, Ali Sigaroudi^3,4^, Philippe Schmitt-Kopplin^1,5*^, Bernhard Michalke^1^

^1^ Helmholtz Zentrum München, Analytische BioGeoChemie, Ingolstädter Landstr. 1, 85764 Neuherberg, Germany

^2^ Uniklinik Köln, Klinik und Poliklinik für Neurologie, Kerpener Str. 62; 50937 Köln

^3^ Universitätsspital Zürich, Klinik für Klinische Pharmakologie und Toxikologie, Rämistr. 100, 8091 Zürich

^4^ Uniklinik Köln, Institut I für Pharmakologie, Zentrum für Pharmakologie, Gleueler Str. 24, 50931 Köln

^5^ TU Munich, Chair of analytical food chemistry, Science center Weihenstephan, Freising, Germany

^¶^Equally contributed

*Corresponding authors:
Desiree Willkommen, Email: desiree.willkommen@helmholtz-muenchen.de

Marianna Lucio, Email: marianna.lucio@helmholtz-muenchen.de

Philippe Schmitt-Kopplin, Email: schmitt-kopplin@helmholtz-muenchen.de

Supporting Information

Table of contents

Table S1 Raw data of non-targeted metabolomic investigation in CSF

Table S2 important masses which reveal a statistical significance

Table S3 Most important neutral masses to distinguish between Parkinson´s disease and controls with all possible assignments to respective masses

Table S4 Summary of network analysis

Table S5 Balanced Error Rate (BER)

S3 Table. Most important neutral masses to distinguish between Parkinson´s disease and controls with all possible assignments to respective masses

| **mass** | **compound** | **All possible assignments** |
| --- | --- | --- |
| 129.04261 | 5-Oxoproline | 4-Oxoproline; L-1-Pyrroline-3-hydroxy-5-carboxylate; 1-Pyrroline-4-hydroxy-2-carboxylate; N-Acryloylglycine |
| 163.09980 | N-Acetylphenylethyl-amine | R-2-Methylimino-1-phenylpropan-1-ol; EPA |
| 172.14637 | Decanoic acid | p-methan-3,8-diol; Ethyl octanoate; 2-methyl-nonanoic acid; 3-methyl-nonanoic acid; 7-methyl-nonanoic acid; 8-methyl-nonanoic acid |
| 188.01433 | p-cresol sulfate | 4-sulfobenzyl alcohol |
| 188.14116 | 10-Hydroxydecanoic acid | 3-hydroxydecanoic acid; 2-hydroxydecanoic acid; 4-hydroxydecanoic acid; 5-hydroxydecanoic acid; 6-hydroxydecanoic acid; 7-hydroxydecanoic acid; 9-hydroxydecanoic acid; 6-hydroxy-3,7-dimethyloctanoic acid |
| 192.06343 | Quinic acid | Valiolone; 2D-5-O-Methyl-2,3,5/4,6-pentahydroxycyclohexanone |
| 210.07402 | Sedoheptulose | Mannoheptulose |
| 234.16207 | Valerenic acid | Bakkenolide A; Confertifolin; Drimenin; Eremophilenolide; Helminthosporal; Hydroxyisopatchoulenone; Polygodial; Sclerosporin; Tetradymol; Curcumenol; Dehydrocurdione; alpha-Cyperone ; (4S,5S)-(+)-Germacrone 4,5-epoxide; 13-Hydroxygermacrone; Curcumenone; Sugeonol; Chrysantherol; 1,13-Dihydroxy-herbertene; 12-Hydroxy-chiloscyphone; Macrophyllic acid A; Curmadione; Onitin; Alcyopterosins O |
| 260.02032 | D-Glucose-6-sulfate | D-galactose-6-sulfate |
| 268.07956 | α-mannosylglycerate | 3-Deoxy-D-glycero-D-galacto-2-nonulosonic acid |
| 304.24043 | Arachidonic acid | Acutilol A; 2-Ketoepimanool; Taxa-4(20),11(12)-dien-5alpha,13alpha-diol; Methandriol; Metholone; 3alpha-Hydroxy-2alpha-methyl-5alpha-androstan-17-one; 17-Methyl-5alpha-androst-2-ene-1alpha; 17beta-Hydroxy-4alpha-methyl-5alpha-androstan-3-one; 3beta-Methoxyandrost-5-en-16beta-ol; Cis-8,11,14,17-Eicosatetraenoic acid; Mesterolone; 18-methyl-5Z,8Z,11Z,14Z-nonadecatetraenoic acid; 17:4(2E,4E,9E,11E)(8Me[R],10Me,15Me[R]); 8R,10,15R-trimethyl-2E,4E,9E,11E-heptadecatetraenoic acid; 4,8,12,16-eicosatetraenoic acid; 6,10,14,18-eicosatetraenoic acid; 4,7,10,13-Eicosatetraenoic acid; 4Z,8Z,11Z,14Z-eicosatetraenoic acid; 5,11,14,17-Eicosatetraenoic acid; Juniperonic acid; 8Z,11Z,14Z,18Z-eicosatetraenoic acid; 7,13-Eicosadiynoic acid; 8,11-Eicosadiynoic acid; 10,13-Eicosadiynoic acid; 2E,8Z,11Z,14Z-Eicosatetraenoic acid; 5Z,13Z,16Z,19Z-eicosatetraenoic acid; 7R,10,13S-trimethyl-2E,4E,9E,11E-heptadecatetraenoic acid; 3,5S,15-trimethyl-7-methylene-2E,9E,11E-hexadecatrienoic acid; 8-[3]-ladderane-octanoic acid; (+)-Serradiol; (-)-Cladielline; (-)-Amijiol; (-)-Isoamijiol; Isotrinervidiol; (-)-Reiswigin A; Mestanolone |
| 306.25612 | Dihomo-γ-linolenic acid | 2,7,11-Cembratrien-4,6-diol; 2alpha-Methyl-5alpha-androstane-3alpha,17beta-diol; 2alpha-Methyl-5alpha-androstane-3beta; 17alpha-Methyl-5alpha-androstane-3alpha,17beta-diol; 17-Methyl-5alpha-androstane-11beta,17beta-diol; Icosatrienoic acid; 5,8,11-Eicosatrienoic acid; 18-methyl-8Z,11Z,14Z-nonadecatrienoic acid; ETrE(11Z, 14Z, 17Z); 2E,4E,8Z-eicosatrienoic acid; Sciadonic acid; 7,10,13-Eicosatrienoic acid; 7,11,14-Eicosatrienoic acid; 8Z,12E,14Z-eicosatrienoic acid; (5Z,9E,14Z)-icosa-5,9,14-trienoic acid; eicosa-5Z,8Z,14Z-trienoic acid; 5Z,13Z,16Z-eicosatrienoic acid; Oncobic acid; Plaunotol; 3Z,6Z,9Z-Octadecatrienyl acetate; 9Z,12Z,15Z-Octadecatrienyl acetate |
